# Genotypic Stability of Lactic Acid Bacteria in Industrial Rye Bread Sourdoughs Assessed by ITS-PCR Analysis

**DOI:** 10.3390/microorganisms12091872

**Published:** 2024-09-11

**Authors:** Liis Lutter, Aljona Kuzina, Helena Andreson

**Affiliations:** Chair of Food Science and Technology, Institute of Veterinary Medicine and Animal Sciences, Estonian University of Life Sciences, Kreutzwaldi 56/5, 51006 Tartu, Estonia; liis.lutter@emu.ee (L.L.);

**Keywords:** rye sourdough, wheat sourdough, LAB genotypes, *L. plantarum*

## Abstract

Sourdough bread production relies on metabolically active starters refreshed daily with flour and water. The stability of sourdough microbial strains is crucial for consistent bread quality. However, many bakeries lack information on the persistence of starter cultures in ongoing sourdough production. Consequently, there is growing interest in identifying microbial strains from regularly used sourdoughs that possess good functional properties and resist changes in the complex growth environment. This study aimed to evaluate the composition and stability of lactic acid bacteria (LAB) in industrial wheat (WS) and rye (RS) sourdoughs propagated over a long period. LAB isolates (n = 66) from both sourdoughs, sampled over four seasons, were identified using phenotypic methods and genotyped via ITS-PCR and ITS-PCR/*Taq*I restriction analysis. Eight LAB species were detected, with *Lactiplantibacillus plantarum* being the most dominant and stable. Nineteen distinct LAB genotypes were observed, highlighting significant diversity. The presence of identical LAB genotypes in both sourdoughs suggests microbial transfer through the environment and bakery workers. LAB in RS were found to be more stable than those in WS. These findings underscore the importance of monitoring microbial stability and diversity in industrial sourdough production to maintain consistent bread quality.

## 1. Introduction

The microbial community and stability of the sourdough greatly affect the quality of bread [1,2,3,4]. Microbial consortia vary among different sourdoughs and baking environments and can shift within a single sourdough over time [5,6,7]. Bakery industries often highlight the distinctive regional characteristics of their bread, attributing its unique qualities to microbes originating from the production environment [6,8,9]. More than 70 lactic acid bacteria (LAB) species have been identified in the sourdough ecosystem, primarily from the family *Lactobacillaceae* [7,10,11]. The most common LAB in sourdough microbiota are *Levilactobacillus brevis*, *Limosilactobacillus fermentum*, *Lactiplantibacillus plantarum*, *Companilactobacillus paralimentarius*, and *Fructilactobacillus sanfranciscensis*, irrespective of the type of flour or inclusion of starter cultures that lack sufficient robustness [7,12]. Some sourdoughs also harbor less predominant species from the genera *Pediococcus*, *Leuconostoc*, and *Weissella*, as well as subdominant species from *Lactococcus*, *Enterococcus,* and *Streptococcus* [8,13]. The occurrence of subdominant populations may be attributed to the abundant growth substrates, which allow the creation of different microbial consortia [8,10,12,14,15,16].

The dominance of obligately heterofermentative LAB, primarily lactobacilli, in both wheat and rye sourdoughs is associated with their ability to adapt to carbohydrate metabolism, amino acid conversion, and response mechanisms to acidic stress [7,17,18]. Despite variations in the chemical composition of wheat and rye flour [19,20], which influence the community dynamics and metabolic kinetics of sourdough fermentation, there are no significant differences in their sourdough microbial community [8]. The ability of microbes to adapt to a particular growth substrate is highly strain-specific; thus, even small changes in substrate quality may affect the sourdough microbiota [21], apart from inherently resilient microorganisms [22]. The prevalence of *L. plantarum*, *L. brevis*, *F. sanfranciscensis*, *L. fermentum*, and *L. mesenteroides* has often been described in wheat sourdoughs, while *L. plantarum*, *L. brevis*, *L. fermentum*, and *F. sanfranciscensis* are common to rye sourdoughs. Additionally, some studies [8,23,24,25] have reported several uncommon LAB species, mainly homofermentative *Lactobacillus helveticus* and *Lactobacillus amylovorus*, in rye bread sourdoughs. Recently, the following subdominant species of lactobacilli, such as *L. diolivorans*, *L. gallinarum*, *L. kimchii*, *L. otakiensis*, *L. parabrevis*, and *L. xiangfangensis*, have been identified in rye sourdoughs, which had not been previously recognized [26]. Frequently, once a new LAB species is detected in sourdoughs, no further details about its phenotypic diversity are given. Several lactobacilli present in sourdoughs are characterized as resilient and resistant in flours and grains, which partly explains the dominance of the same *Lactobacillus* species in sourdoughs [10]. As stated by Meroth et al. [27], *F. sanfranciscensis* and *L. fermentum* are competitive lactobacilli in rye flour and become dominant during sourdough fermentation [18]. According to Gänzle and Vogel [28], the dominance of one microbial species and/or strain in sourdough can also be attributed to interaction between microbes, such as the formation of organic acids, but also other specific antimicrobial substances like bacteriocins, for instance, reutericyclin by *L. reuteri* or plantaricin by *L. plantarum* [23,29].

Some bakeries are known to use spontaneously initiated sourdoughs as they are metabolically active, with continuous renewal for even more than 100 years [30,31]. The microbiota of traditional sourdoughs can maintain stability for years, and occasionally for decades [5,27,28,32,33,34]. However, some studies [6,22,35] emphasize the instability of the sourdough ecosystem even over relatively short periods. The stability of technological process parameters (principally fermentation temperature and time, back-slopping, dough yield and pH) over time is essential in ensuring the stability of the sourdough microbiota, as the constant application of the same technological parameters can favor the dominance of microbial strains better adapted to the environmental conditions [31,36]. At the same time, sourdough stability is also affected by factors such as the adaptability of microorganisms to changing environmental conditions, synergistic and antagonistic interactions between microorganisms, and the microbiota of the bakery environment [5,31]. While the back-slopping of sourdough generally selects heterofermentative lactobacilli, the number of refreshment steps as well as their frequency and duration affect the dynamics and stability of the sourdough microbial communities [6]. The number of refreshment stages determines the initial pH of the fermentation and thus affects the growth and extent of LAB fermentation [37,38].

The growing trend of using traditional sourdough in bread making underscores the need for additional research. So far, information on the characterization of traditional Estonian rye sourdough remains limited, although the rye bread is a staple food in this region. Previous studies have focused on mapping the microbial community of laboratory-initiated and -propagated sourdoughs or artisan sourdoughs rather than industrial sourdoughs. This study aims to evaluate the stability and variation of LAB in two industrial sourdoughs used for rye bread making, one based on rye flour and the other on wheat flour, collected from an Estonian bread manufacturer throughout the year. The rye sourdough has been maintained for over 40 years, while the wheat sourdough has been periodically refreshed. Despite their long-term use, there is a lack of precise information about the microbial communities and the stability of these starters, highlighting the need for this research. Moreover, the findings of this research can aid in optimizing fermentation processes, developing robust starter cultures, and enhancing bread quality.

## 2. Materials and Methods

### 2.1. Isolation of Lactic Acid Bacteria from Sourdough Samples

Sourdough LAB were isolated from both wheat and rye sourdoughs obtained from an Estonian bread manufacturer. The rye sourdough (RS) starter was created over 40 years ago using an unknown commercial LAB starter culture and has been refreshed daily up to the present day. The wheat sourdough (WS) was also prepared in a separate room by fermenting a flour–water mixture with an unknown commercial LAB strain. Unlike the RS, the WS is periodically renewed at 5- to 10-year intervals; at the time of sample collection, it was 4 years old. The WS was fermented at 20–24 °C for 24 h, whereas the RS was fermented at 30 °C for 6 h. Both sourdoughs were continuously fed at 24 h intervals.

Sourdough samples were collected in each season over one year. Before culturing, RS samples (10 g) were homogenized in 90 mL of sterile 0.9% saline solution at 300 rpm for 5 min using a Stomacher^®^ 400 Circulator homogenizer (Seward Ltd., Worthing, UK). Tenfold serial dilutions were prepared from the homogenized material and from the WS samples, which did not require homogenization due to their consistency. Selected dilutions were plated on MRS agar (De Man, Rogosa, and Sharpe Agar, LabM Ltd., Bury, UK) media. The plates were incubated in parallel under aerobic, anaerobic (BD BBL™ GasPak™ System, Becton, Dickinson and Company, Franklin Lakes, NJ, USA), and microaerobic (10% CO_2_) conditions for up to 72 h at 30 °C.

Based on colony and cell morphology, fourteen LAB isolates were selected from the first sampling period (I SP, winter), with six from the WS and eight from the RS. Twelve LAB isolates (seven from WS and five from RS) were chosen from the second sampling period (II SP, spring). Eighteen isolates (seven from WS and eleven from RS) were selected from the third sampling period (III SP, summer). The fourth sampling period (IV SP, autumn) consisted of twenty-two LAB isolates (nine from WS and thirteen from RS). All microbial isolates (n = 66) were stored in MRS broth (Biolife Italiana, Monza, Italy) with 50% glycerol at −80 °C until analysis.

### 2.2. Identification of LAB Isolates

All LAB isolates were cultivated on MRS agar plates and incubated for 48 h at 37 °C according to the oxygen consumption requirements of the isolate, either in an aerobic, anaerobic, or 10% CO_2_ environment. The purity of the selected colonies was verified by microscopic examination of Gram-stained preparations. For biochemical identification of LAB, the API^®^ 50 CH kit (bioMérieux SA, Paris, France) test together with a search of the APIWEB™ database (bioMérieux SA, Paris, France) was performed according to the manufacturer’s protocol. Additionally, MALDI-TOF mass spectrometry (MALDI Biotyper, Bruker Daltonics, Bremen, Germany) was used, with this analysis carried out at the Tartu Veterinary and Food Laboratory.

### 2.3. DNA Extraction from LAB Isolates

Grown LAB colonies were collected from MRS plates using a 10 µL inoculating loop for isolating bacterial DNA. Colonies were transferred to 1.5 mL microcentrifuge tubes containing MRS broth and incubated at 37 °C for 24 h. DNA isolation was performed using the QIAamp DNA Mini Kit (QIAGEN, Venlo, The Netherlands) with modifications to the manufacturer’s instructions as follows: 500 μL of the pre-incubated pure culture in MRS broth was combined with 100 μL of enzyme mixture, consisting of 50 µL of lysozyme (10 mg mL^−1^, Alfa Aesar, Ward-Hill, MA, USA), 15 µL of mutanolysin (10 KU mL^−1^, Sigma Aldrich, St. Louis, MO, USA), and 35 µL of TE50 buffer (10 mm Tris pH 8.0, 1 mm EDTA, 50 mm NaCl). This mixture was added to a sterile 2 mL tube containing 0.1 mm glass beads. The tubes were incubated for 1 h at 37 °C, then centrifuged at 2100 rpm for 1 min. The subsequent DNA isolation steps followed the manufacturer’s instructions. DNA samples were stored at −20 °C.

### 2.4. PCR Amplification of 16S rDNA

All DNA samples were subjected to PCR for bacterial 16S rDNA gene analysis using the universal primers 27F (5′-AGA GTT TGA TCM TGG CTC AG-3′) and 1492R (5′-TAC GGY TAC CTT GTT ACG ACT T-3′). The PCR reaction mixture consisted of 1X Titan Taq 5× PCR Mix Ready-to-Load (Bioatlas OÜ, Tartu, Estonia), 0.15 µm each primer, 2 ng µL^−1^ template DNA, and ultrapure water to a final volume of 20 µL. The PCR temperature profile was as follows: enzyme activation at 94 °C for 5 min, followed by 30 cycles of denaturation at 94 °C for 60 s, annealing at 60 °C for 60 s, and elongation at 72 °C for 90 s, concluding with a final elongation step at 72 °C for 10 min. All PCR reactions (including ITS-PCR) were performed in a ProFlex™ thermocycler (3 × 32-well PCR System, Applied Biosystems, Waltham, MA, USA).

### 2.5. ITS-PCR and Restriction with TaqI

ITS-PCR was performed with the primers L1 (5′-CAA GGC ATC CAC CGT-3′) and G1 (5′-GAA GTC GTA ACA AGG-3′), which are complementary to the conserved regions of 16S and 23S rDNA and have been successfully used in a previous study by Dec et al. [39]. These primers amplify the variable spacer regions between the 16S and 23S rDNA genes, which can vary in length and sequence among different bacterial species, providing a useful marker for differentiation. PCR reactions were similar to those described for the 16S rDNA analysis using primers 27F and 1492R, with the final volume being 40 µL and the primers’ concentration being 0.6 µm each. The PCR conditions were as follows: an initial incubation at 95 °C for 60 s, then 35 cycles of denaturation at 95 °C for 60 s, annealing at 55 °C for 60 s, and extension at 72 °C for 2 min, with final elongation at 72 °C for 7 min. 

ITS-PCR products (10 µL) were digested with FastDigest *Taq*I restriction enzyme (Thermo Fisher Scientific Baltics UAB, Vilnius, Lithuania) for 10 min at 65 °C.

### 2.6. Gel Electrophoreses

The PCR, ITS-PCR, and ITS-PCR/*Taq*I restriction products (10 µL) were evaluated by gel electrophoresis, for which a 1.8% (2.5% in case of restriction products) agarose gel was prepared in 1× TBE (Tris-borate-EDTA, Bioatlas OÜ, Tartu, Estonia) buffer. For each electrophoresis, 1 kb (Bioatlas OÜ, Estonia) and 100 bp (Solis BioDyne OÜ, Tartu, Estonia) DNA Ladder size markers were used to estimate DNA size. Electrophoresis was carried out for 30 min at a constant voltage of 110 V. Amplified DNA products were visualized under UV radiation with a UVsolo touch (Analytik Jena, London, UK) gel visualization device.

### 2.7. Cluster Analysis

The visualized DNA segments were analyzed and compared using the VisionWorks Acquisition and Analysis Software (ver: 8.20.17096.9551, Analytik Jena, London, UK), and a general dendrogram was constructed based on the pattern of ITS-PCR/*Taq*I products. The unweighted pair group method average (UPGMA) method based on Jaccard’s similarity was used to create the dendrogram, with the cut off value of 0.5.

The molecular results were compared with those obtained from API and MALDI-TOF analyses, as well as with the patterns of the following reference strains: *L. plantarum* ATCC 14917 (obtained from BioCC OÜ, Tartu, Estonia), *L. plantarum* TAK59 (Nordwise Biotech OÜ, Tartu, Estonia), *L. plantarum* LB-1 (Chr. Hansen, Hørsholm, Denmark), *L. fermentum* ME-3 (VF Bioscience, Loos, France), *L. paracasei* SEMAC3 (identified previously by MALDI-TOF analysis), and *L. rhamnosus* GG (LGG^®^ Gefilus^®^, Valio OY, Helsinki, Finland). Reference strains of LAB were also used as positive control samples for PCR and ITS-PCR analyses.

## 3. Results and Discussion

### 3.1. LAB Species in Sourdoughs

Eight species of LAB were identified using MALDI-TOF analysis (Table 1): *Lactiplantibacillus plantarum* 33 (50%), *Lentilactobacillus parabuchneri* 8 (12%), *Lacticaseibacillus paracasei* 7 (11%), *Limosilactobacillus fermentum* 5 (8%), *Companilactobacillus mindensis* 3 (5%), *Companilactobacillus paralimentarius* 3 (5%), *Schleiferilactobacillus harbinensis* 1 (2%), and *Weissella cibaria* 1 (2%). Four LAB isolates could only be identified at the genus level by MALDI-TOF: three *Lactobacillus* sp. (5%) and one *Pediococcus* sp. (2%). One rye sourdough (RS) isolate (2%) remained unidentified by MALDI-TOF.

Discrepancies between the API kit and MALDI-TOF results were observed in 28 (42%) of the LAB isolates, likely due to limitations within the APIWEB™ database. For instance, four isolates identified by MALDI-TOF as *L. parabuchneri* were identified as *L. buchneri* by API analysis. Additionally, six species were identified solely by API analysis and were not verified by MALDI-TOF. The best match between these two biochemical identification methods was with *L. plantarum*, where 28 out of 33 isolates (85%) were identified concordantly.

Although numerous LAB species have been identified in sourdoughs, typically only two to three dominate in mature sourdoughs [40,41]. The most frequently identified LAB species in this study was *L. plantarum*, comprising 50% of all isolates. This species dominated both wheat (31%) and rye sourdoughs (65%), and was present in all four sampling periods (SPs). The results are consistent with previous studies [10,26,42,43] that confirm *L. plantarum* as one of the most common LAB in sourdoughs. *L. plantarum* is primarily associated with plant materials (e.g., cereal and legume flours) and is involved in numerous food fermentations, including sourdough [44,45,46,47,48]. Its versatile metabolism and high adaptability to complex and diverse environmental conditions (e.g., high acid tolerance) are due to its extensive genomic capacity for carbohydrate metabolism and regulatory mechanisms [49,50]. Additionally, *L. plantarum* displays significant antimicrobial activity, producing a range of antimicrobial compounds such as organic acids, hydrogen peroxide, and bacteriocins like plantaricins, which can inhibit various pathogenic bacteria and fungi [50,51,52]. Because of these metabolic traits, *L. plantarum* effectively competes with other native species and/or biotypes of the same species in cereal flour [22,45]. Plantaricins have also shown activity against other *L. plantarum* strains [53] and *L. sanfranciscensis* strains [54]. Therefore, when using a single strain of *L. plantarum* as a starter culture for initiating sourdough fermentation, it is important to ensure that the selected strain demonstrates resilience against autochthonous LAB species, particularly various strains of *L. plantarum*.

After *L. plantarum*, the other most frequently identified LAB species also belonged to the heterofermentative LAB group, including *L. parabuchneri*, *L. paracasei*, and *L. fermentum*. *L. parabuchneri* and *L. paracasei* were present in all SPs except the first (I SP), while *L. fermentum* was undetected in the fourth sampling period (IV SP). *L. plantarum* and either *L. brevis* or *L. fermentum* are often found together in many food fermentations, including spontaneous sourdoughs [55,56,57]. Bessmeltseva et al. [58] identified four dominant LAB species in spontaneously started rye sourdoughs after 56 days of propagation in a controlled laboratory environment: *L. plantarum*, *L. brevis*, *C. paralimentarius*, and *Lactobacillus crustorum*. They observed changes in the proportions of LAB communities after 42 propagation cycles, concluding that the instability of the sourdough LAB community may be the low level of sourdough-specific LAB in rye flour. In the current research, *L. parabuchneri* and *L. paracasei* were more common in WS, similar to the findings of Fraberger et al. [26]. A high prevalence of *L. fermentum* in sourdough has also been observed [7,10,59,60], as it is characteristic of the microbial community of rye flour and can become the primary species during sourdough fermentation [27]. Gaglio et al. [61] confirmed the remarkable resilience of *L. brevis* to high-stress conditions, which supports this species’ adaptability in sourdough microbiota. It has also been noted that *F. sanfranciscensis* typically dominates in traditionally prepared and older sourdoughs, while *L. plantarum* and *L. brevis* are predominant in younger sourdoughs; as sourdough reaches maturity, the diversity of LAB generally decreases [56]. Contrary to other studies, *L. parabuchneri* has been less commonly found in sourdoughs [10], although it was the second most frequently identified species among the LAB isolates in this study.

The remaining LAB species, such as *C. paralimentarius* and *S. harbinensis*, were less frequently detected in the sourdough samples. *C. paralimentarius* was present in all SPs except the I SP, while *S. harbinensis* was detected only in the III SP and only in RS. According to Bessmeltseva et al. [58], *C. paralimentarius* is also a subdominant species in RS. However, this species has been frequently reported in various sourdough studies [57,62], particularly in Greek and Belgian sourdoughs. In some sourdoughs, *C. paralimentarius* has even been identified as the dominant species [63,64]. Therefore, it can be concluded that if *C. paralimentarius* is present in cereal flour in higher abundance, it can compete with other LAB and potentially become one of the dominant species in sourdough fermentation.

*Schleiferilactobacillus harbinensis* (formerly *Lactobacillus harbinensis*) is a novel LAB species detected by Sekwati-Monang and Gänzle [65] in sorghum sourdough, by Lim et al. [66] in rice sourdough, and later by Rappaport et al. [67]. This species has been identified in various traditional fermented foods [68,69,70,71], but it is not commonly found in sourdough microbiota. To date, the occurrence of another species from the genus *Schleiferilactobacillus*, such as *S. perolens*, has also been revealed in sourdough [72], and this species is closely related to *S. harbinensis* [73]. *S. harbinensis* has shown promising antifungal activity in dairy products, making it suitable as a bioprotective starter culture [74,75,76]. Consistent with the metabolic pathways for hexose fermentation, the primary metabolite of *S. harbinensis* in both wheat- and sorghum-based sourdoughs is lactate [65].

A meta-analysis of 583 back-slopped sourdoughs by Van Kerrebroeck et al. [13] identified the presence of *Weissella* species (e.g., *Weissella cibaria*, *Weissella confusa,* etc.) in 15% of the studied sourdoughs. In the current study, *W. cibaria* was detected once in RS during the I SP. Ispirli et al. [77] also identified *W. cibaria* and *W. confusa* in RS. *W. cibaria* is known to be a predominant species in sourdoughs, often observed in both Type I and Type II sourdoughs, as *Weissella* species can grow well across a wide range of temperatures (at 15–37 °C), water activity, and pH [78,79]. Studies of *Weissella* spp. have primarily focused on exopolysaccharide production and characterization. For instance, *W. cibaria* is known for its ability to produce homopolysaccharides called dextrans, which improve the softness of fresh bread [80].

In a study by Boreczek et al. [81], *Weissella* and *Leuconostoc* bacteria were isolated from samples collected after 24 h of fermentation. However, by 72 h, *Weissella* was no longer detected in wheat, spelt, or rye sourdoughs when analyzed using classical microbiological methods. Sequencing results initially showed that after 24 h, the abundance of *Weissella* was higher than that of *Lactobacillus*. As fermentation progressed, however, the abundance of *Weissella* declined to 11% after 48 h and to just 5% after 72 h. This phenomenon is likely due to dynamic shifts in the microbial community during the sourdough fermentation process. Initially, *Weissella* and *Leuconostoc* thrive and dominate due to their ability to grow rapidly and tolerate early fermentation conditions. As the fermentation environment changes due to factors such as pH reduction and the accumulation of fermentation by-products, the microbial community shifts, favoring other LAB, such as *Lactobacillus* species, which are more acid-tolerant and can outcompete *Weissella* and *Leuconostoc* in the later stages.

It is well known that the development of the sourdough ecosystem can be categorized into three phases [11,38,45]. In the first phase, non-specific microbes, such as species from the genera *Enterococcus*, *Lactococcus*, and *Leuconostoc*, dominate the sourdough. Subsequently, in the second growth phase, LAB specific to sourdough, such as species from the genera *Lactobacillus*, *Pediococcus*, and *Weissella*, surpass other species. Finally, in the third phase, only well-adapted LAB, such as *L. plantarum* and *L. fermentum*, dominate the sourdough ecosystem [31,38]. Consistent with these phases, our findings revealed that *L. plantarum* was the most frequently identified species across all sampling periods, confirming its role as a dominant and well-adapted LAB in mature sourdoughs. Additionally, the presence of species like *L. fermentum* further supports the progression towards a more specialized and resilient microbial community as fermentation advances.

### 3.2. Diversity and Stability of LAB Genotypes

**Figure 1:**
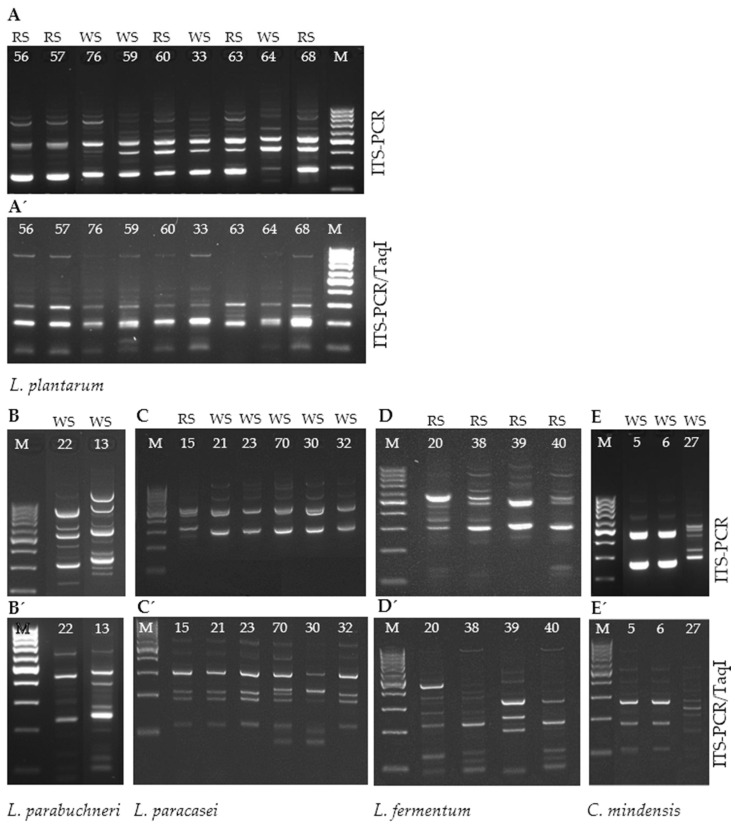
Example of ITS-PCR of LAB species from different sourdoughs (**A**–**E**) and the restriction of the same ITS-PCR products with the *Taq*I enzyme (**A′**–**E′**). M—100 bp DNA ladder; WS—wheat sourdough; RS—rye sourdough; numbers—sample ID numbers. The patterns show the differentiation of LAB species and strains, including *L. plantarum* (**A**,**A′**), *L. parabuchneri* (**B**,**B′**), *L. paracasei* (**C**,**C′**), *L. fermentum* (**D**,**D′**), and *C. mindensis* (**E**,**E′**) across various sourdoughs.

The biochemical identification of LAB was further validated through molecular analysis. Subsequent investigations relied primarily on MALDI-TOF results, as the subjective interpretation of color changes in API tests and the limited scope of its database present notable constraints. PCR and ITS-PCR analysis confirmed the presence of bacterial 16S and 23S rDNA in the samples. The ITS-PCR products amplified with the L1/G1 primers, along with those generated by subsequent digestion with the *Taq*I restriction enzyme, were clearly distinguishable, effectively differentiating LAB isolated from sourdoughs at both the species and strain levels (Figure 1).

The gel electrophoresis results show that the ITS-PCR/*Taq*I patterns of the *L. plantarum* isolates from the III SP (sample no. 60) and the IV SP (all other samples) were highly similar (Figure 1A′). In contrast, the ITS-PCR and ITS-PCR/*Taq*I patterns of *L. parabuchneri* isolates from the II SP (no. 22) and III SP (no. 13) were distinctly different (Figure 1B′). *L. paracasei* and *L. fermentum* isolates, which appeared similar based on the ITS-PCR pattern (Figure 1C,D), were revealed to be completely different after *Taq*I digestion (Figure 1C’,D’). Additionally, the uncut and cut ITS-PCR patterns of *C. mindensis* displayed noticeable differences between two genotypes (samples 5 and 6 versus sample 27; Figure 1E′), even though all samples originated from the same sampling period and sourdough.

The ITS-PCR and ITS-PCR/*Taq*I product sizes were analyzed using VisionWorks software. Based on species and banding patterns, the isolates were grouped into eight main genotypes (Table 2).

In the studied sourdoughs, six *L. plantarum* (A1, A2, A5, A6, A9, and A11) and two *L. paracasei* genotypes (C1 and C2) were detected at least twice over the course of half a year, indicating their persistence in the sourdough environment. Furthermore, nine genotypes (A2, A3, A4, A6, A7, A8, A11, C2, and F2) were found in both WS and RS, suggesting potential microbial transfer between these sourdoughs within the bread industry. This observation aligns with Scheirlinck et al. [5], who identified sourdough-specific species, primarily *F. sanfranciscensis*, in the air of bread industry storage and workrooms, on equipment surfaces, and workers’ hands, indicating these species can spread throughout the propagation environment. Moreover, genetically identical isolates of *L. plantarum*, *L. spicheri*, and *F. sanfranciscensis* were identified in both sourdough and bakery environment samples, implying that certain strains dominate both in sourdoughs and in their associated propagation environments. These strains were observed to persist in sourdoughs for at least three years [5]. This may explain the results of the present study, where LAB of the same genotype were detected in both WS and RS, despite these sourdoughs being kept separate in the industry.

Previous studies by Spicher and Schröder [82], Böcker et al. [32], and Gänzle et al. [83] have confirmed that sourdough composition can remain stable at the strain level for at least two decades when traditional propagation methods are employed. Rosenquist and Hansen [23] reported only minor changes in the microbiota of two industrial sourdoughs during a 7-month investigation. In contrast, Böcker et al. [84] observed significant shifts in the microbial composition of sourdough made with rye sourdough extract over a 10-year period. Despite these changes, strains of *L. reuteri* with similar physiological properties and molecular patterns were consistently isolated at each sampling point [28,84], underscoring the robustness and stability of certain LAB strains over extended timescales. Gänzle and Vogel [28] later attributed the long-term stability of *L. reuteri* in sourdoughs to its production of reutericyclin, a low-molecular-weight antibiotic that selectively inhibits other LAB, such as *F. sanfranciscensis,* while enabling *L. reuteri* and other species like *L. amylovorus*, *L. pontis* and *L. frumenti* to persist.

During the entire experimental period, a total of 18 different LAB genotypes were identified in WS, while 21 LAB genotypes were found in RS. Five LAB genotypes (A1, A5, A6, A9, and C2) remained stably present in RS for at least six months. In contrast, no LAB genotypes were consistently present in WS, indicating greater stability of LAB in RS. The significant microbial diversity observed can be attributed to the age of the sourdoughs and the conditions of the industrial production environment. Since both sourdoughs undergo daily back-slopping in an open production system, it is likely that new microbial strains enter from raw materials and the bakery environment.

The highest number of genotypes in RS was observed during the III and IV SPs, with 10 and 8 genotypes, respectively. This could be linked to higher external temperatures, which likely influenced the temperature in the bakery’s production rooms. In WS, the number of genotypes ranged from four to six across the SPs, peaking at six during the last sampling in autumn. These findings align with other studies suggesting that environmental microbiota, along with the stability of technological parameters and ingredients, influence sourdough microbiota stability [5,12,14,85,86].

Vrancken et al. [87] showed that under laboratory conditions, the sourdough microbiota is primarily shaped by raw materials like flour. Reese et al. [86] found a substantial overlap between the microbial communities in sourdough starters and those on bakers’ hands, with approximately 26% of all amplicon sequence variants in the starters being common to both but absent from the flour. Most microbial taxa from the flour successfully colonized some sourdough samples but were not present on bakers’ hands. Additionally, Minervini et al. [60] explored LAB and yeast contamination from house microbiota during sourdough back-slopping, confirming that dominant sourdough species also dominate the house microbiota. *Lactobacillus* showed the highest capacity to colonize bakery equipment and sourdough compared to genera such as *Bacillus*, *Paenibacillus, Staphylococcus*, and *Enterococcus*. However, *Lactobacillus* species varied in adaptability; for example, *F. sanfranciscensis* was found in all samples and bakeries, while *L. plantarum* was consistently present in flour at lower abundances, leading to its less frequent detection in the environment. Muthappa et al. [88] also identified the seasonal transfer of environmental bacteria to the food matrix in bakeries.

In Bauer Munch-Andersen’s study [89], it took 11–15 days for legume-based sourdoughs to reach a stable pH and microbial community, a longer period compared to the 5–7 days reported for many cereal-based sourdoughs [38,90] under daily propagation. This extended stabilization period may be due to the unusually large proportion of sourdough used during the refreshing phase (50% *w*/*w*). While species stability was achieved in mature sourdoughs, strain stability was not assessed. Notably, *L. plantarum* and *P. pentosaceus* dominated at fermentation temperatures of 30 °C and 22 °C, respectively. Sourdoughs fermented at 22 °C exhibited greater microbial diversity than those fermented at 30 °C, likely due to the inhibitory effect of higher temperatures on microbial diversity [89]. In the current study, *L. fermentum* strains were more prevalent in RS, which fermented at higher temperatures than WS. This aligns with literature stating that the optimal growth temperature for *L. fermentum* is 30–37 °C [91]. However, further research is needed to determine the precise impact of temperature on the growth of these strains.

Galli et al. [92] studied LAB robustness during ten days of sourdough propagation, finding that stable microbial communities were established after seven refreshment steps, although species exhibited varying levels of competitiveness. *L. farciminis* Lf19, a species uncommon in sourdough, was highly competitive, whereas *L. rossiae* Lr9 was less robust. In liquid sourdough (dough yield = 330) and in the presence of baker’s yeast *(Saccharomyces cerevisiae*), *F. sanfranciscensis* showed low competitiveness, likely due to maltose depletion and extended refreshment times.

Among the studied isolates, *L. plantarum* genotypes (A1–A12) were the most prevalent, comprising 33 (54%) of the isolates (Table 2). Most (7/12) genotypes were present in both WS and RS. The reference strain *L. plantarum* LB-1 was 100% similar to genotype A11, *L. plantarum* TAK59 was 50% similar to genotype A9, and *L. plantarum* ATCC 14917 was 20% similar to genotype A2. *L. plantarum* emerged as the most stable and abundant microbial species in terms of both isolates and genotypes, consistent with literature indicating that *L. plantarum* is highly adaptable, capable of reproducing across a wide temperature range and in conditions of carbohydrate deficit [93]. This species can utilize alternative energy sources such as amino acids and nucleotide sugars [12], and demonstrates good resistance to phenolic compounds, which are abundant in organic flours and known to inhibit the growth of most LAB [94]. In traditional sourdough systems, a single phenotype remained dominant throughout the sampling period, while in organic sourdoughs, the phenotype changed after three months [23].

Pepe et al. [95] studied the molecular diversity of *L. plantarum* strains isolated from four naturally fermented Italian artisan sourdoughs and found that thirty *L. plantarum* strains were grouped into ten different genomic groups, with five strains being clearly differentiated. Three of these genomic groups contained isolates from different sources, distinguishable based on their technological characteristics. Similarly, Minervini et al. [22] observed that five out of seven *L. plantarum* strains dominated sourdough samples throughout ten days of propagation, while two other strains were outcompeted by the indigenous LAB from the flour and production environment. These findings underscore the importance of selecting sourdough starter cultures based on both their functional traits and the resilience of the strains.

Most studies have focused on monitoring microbial dynamics in laboratory and artisan sourdoughs over weeks to months, with fewer studies on industrial sourdoughs. Kitahara et al. [96] isolated 57 LAB strains from five wheat and rye sourdoughs, including two from the same manufacturer. *F. sanfranciscensis* was the dominant LAB in all sourdoughs (n = 21), with the isolates clustered into four groups at 80% similarity. Although the *F. sanfranciscensis* strains from the same manufacturer’s sourdoughs showed high similarity, the overall LAB composition exhibited distinct diversity. Research has also indicated that *F. sanfranciscensis* strains in sourdough are not identical, and their ability to adapt varies. For instance, Siragusa et al. [35] found that after several propagations, only three out of nine *F. sanfranciscensis* strains dominated in Type I sourdough. These studies suggest that sourdough samples from different regions contain genetically distinct *F. sanfranciscensis* strains with geographical specificity. The persistence of *F. sanfranciscensis* in sourdough appears to depend on the specific strain, in contrast to *L. plantarum* strains, which have demonstrated greater robustness in outcompeting the indigenous flour microbiota [22]. Similar to Pino [97], we did not find a prevalence of *F. sanfranciscensis*, which is typically expected to have a strong association with *L. plantarum* [22,98].

The results of the current study align with findings from Lhomme et al. [99], which highlight that environmental factors and technological processes contribute to genetic diversity in LAB strains. Genetic analysis of *F. sanfranciscensis* strains from Italian sourdoughs revealed significant diversity, with 22 different pulsotypes and 19 sequence types identified among 24 isolates collected over several years. Notably, three *F. sanfranciscensis* strains, sharing the same pulsotype and sequence type, were isolated from the same processing area over three consecutive years [100]. This suggests that processing conditions and the manufacturing environment play a more critical role in determining strain abundance than geographical location. Similarly, a study on *F. sanfranciscensis* strains from Chinese traditional sourdoughs found no correlation between geographical origin and strain types, as no region-specific strains were identified [101]. Strains from diverse origins often grouped together, further supporting the idea that factors other than geography influence microbial communities.

Several studies [7,8,13,90,102] have confirmed that neither the type of flour nor the geographical location of producers significantly affects the microbial community structure of mature sourdoughs. For instance, Comasio et al. [102] found that the microbial composition of sourdoughs from the same producer remained consistent, regardless of the flour type used. This suggests that house microbiota or processing conditions likely have a more significant impact on microbial community structure than the type of flour.

*L. paracasei* genotype C2 maintained 100% similarity across five isolates over at least two SPs. This genotype also showed 100% similarity with the reference strain *L. paracasei* SEMAC3. The effectiveness of *L. paracasei* strains isolated from dairy products as starter cultures in sourdough bread making has been demonstrated in a few studies [103,104]. Additionally, *L. paracasei* has exhibited inhibitory properties against pathogenic, opportunistic bacterial strains, and fungal strains, highlighting its promising antimicrobial characteristics [42,105]. It has also been shown to reduce levels of the anti-nutrient phytate [104] and to degrade low-fermentable oligo-, di-, and monosaccharides, as well as polyols, during sourdough fermentation, due to the enzyme β-fructosidase FosE [106]. Although *L. paracasei* has been identified in both Type I and Type II sourdoughs [26,42,105,106,107,108,109] and is not a dominant species, it possesses remarkable functional properties, making it a valuable candidate for use as a starter culture in sourdough bread preparation. However, further research is needed to fully understand its robustness in this context.

**Figure 2:**
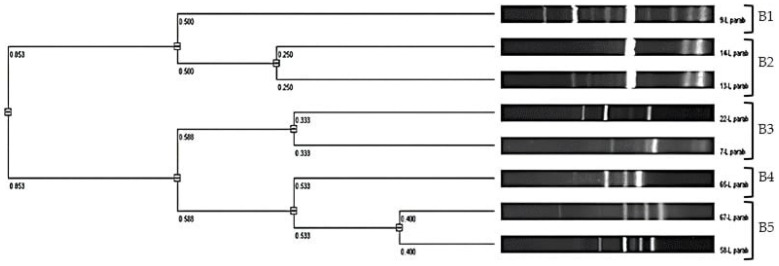
ITS-PCR UPGMA dendrogram of *L. parabuchneri* strains digested with the *Taq*I restriction enzyme, where B1–B5 represent the genotypes of the species.

For the eight *L. parabuchneri* isolates, the cluster analysis (Figure 2) identified five distinct genotypes, showing excellent concordance with the results in Table 2.

*L. parabuchneri* genotypes B1 and B4 originated from the II SP in RS, while the other three genotypes (B2, B3, and B5) were identified in WS during the III, II, and IV SPs, respectively. The similarity among these strains was observed only when samples were from the same SP and the same type of sourdough. This suggests that the strains of this microbial species varied across different seasons.

Each *L. fermentum* isolate was associated with a distinct genotype (Table 2). Genotype D1 was identified in RS during the I SP, while genotypes D2 and D4 were detected in RS during the III SP. Genotype D3, unique to WS, was observed during the II SP. Additionally, genotype D5 was identified in RS during the II SP. Notably, the reference strain *L. fermentum* ME-3 showed 100% similarity to genotype D3.

The genotypic diversity of microbial strains may be influenced by variations in technological and environmental parameters [102]. While traditionally prepared sourdough microbes are generally stable, factors such as the chemical composition of the flour and the presence of bacteriophages can alter the sourdough microbiota [110]. These changes in microbial strains can, in turn, affect the technological properties of the sourdough, as well as the sensory properties and overall quality of the bread [31].

Scheirlinck et al. [5] demonstrated that the LAB composition in wheat and rye sourdoughs remained relatively stable over the course of a year. The sourdoughs were collected from different bakeries, and the authors concluded that the stability of LAB in sourdough is primarily influenced by technological parameters such as temperature, acidity, number of refreshment stages, dough yield, salt content, redox potential, and both fermentation time and temperature. The impact of these endogenous factors on sourdough stability has been further clarified by several authors [31,87,111,112,113,114]. Viiard et al. [25] also found that the stability of RS microbiota is maintained only when bakery technological parameters are carefully controlled.

Both *C. mindensis* genotypes (E1 and E2) originated from the I SP of WS (Table 2). Two isolates of *C. paralimentarius* (F2) were collected during the III SP from both RS and WS, while one isolate from a different genotype (F1) was found during the II SP of WS. Due to the small number of isolates of *C. mindensis*, *C. paralimentarius*, *S. harbinensis*, and *W. cibaria*, it is not possible to draw definitive conclusions about the stability of these strains in the sourdoughs.

**Figure 3:**
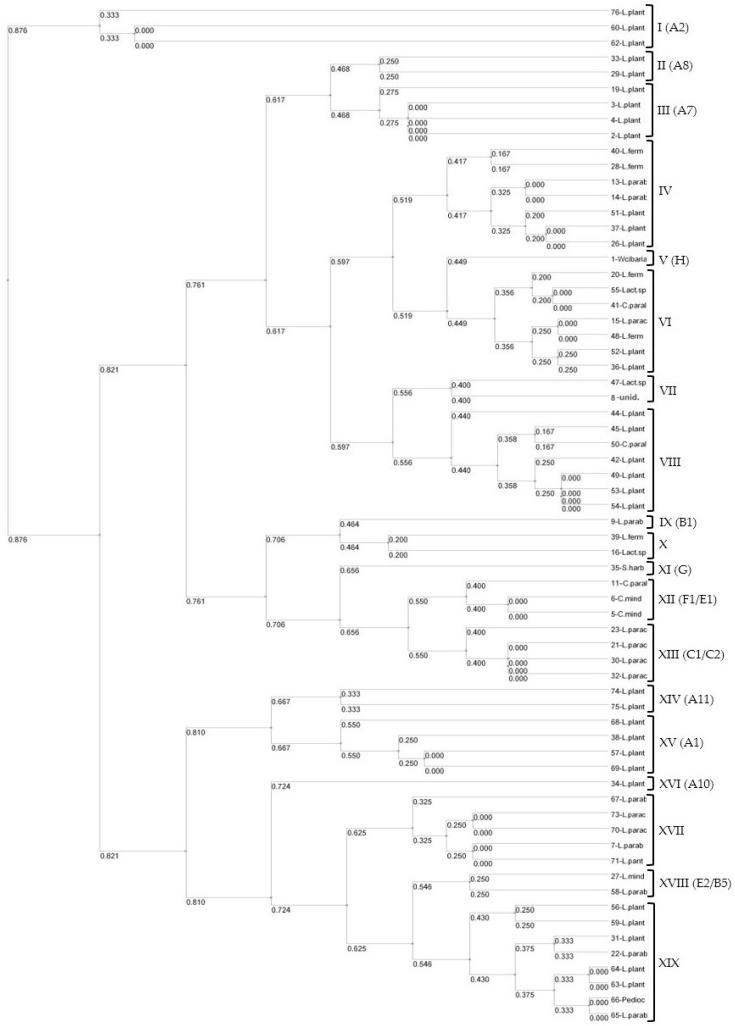
UPGMA dendrogram of LAB (n = 66) based on ITS-PCR products digested with *Taq*I. Each cluster is numbered with Roman numerals with the corresponding genotypes (grouped according to their electrophoresis patterns in Table 2) indicated in parentheses.

Cluster analysis of the 66 LAB isolates identified 19 distinct genotypes (Figure 3) using a cut-off value of 0.5, highlighting the diversity within the LAB community in the studied sourdoughs. Each cluster represents a unique genotype, indicating a wide range of genetic variability among the isolates. The clustering reflects the relationships between isolates based on their ITS-PCR/*Taq*I profiles, with close proximity in the dendrogram suggesting high genetic similarity among certain isolates. The results of the cluster analysis were generally in good concordance with the genotypic characterizations presented in Table 2.

Specifically, a clearly distinct cluster was observed for *L. plantarum* genotype A2 (Cluster I), indicating its unique genetic profile. Other *L. plantarum* genotypes, such as A8, A7, A11, A1, and A10, were also found in separate clusters, underscoring the genetic diversity within this species in the studied sourdoughs. Additionally, clusters IV, VI, VIII, XVII, and XIX were more heterogeneous, comprising isolates from different species and genotypes, reflecting a broader range of genetic variability within these groups despite their overall similarity, which led to their clustering together.

The ITS-PCR/*Taq*I dendrogram analysis enabled the identification of bacteria that MALDI-TOF could not identify or could only identify at the genus level. For instance, sample no. 16 had an 80% similarity to the *L. fermentum* pattern, and sample no. 55 matched *C. paralimentarius* with 100% similarity. Although there were instances where distinct species, such as *L. plantarum*, *Pediococcus* sp., and *L. paralimentarius* (Cluster XIX), were clustered together, the overall strong concordance between the cluster analysis and Table 2 validates the suitability of the genotyping method. This confirms the relative accuracy of the ITS-PCR/*Taq*I method and the reliability of the identified genotypes across different sourdoughs and sampling periods.

## 4. Conclusions

This study demonstrated that *Lactiplantibacillus plantarum* strains were the dominant lactic acid bacteria among the isolates from both wheat and rye sourdoughs. Molecular analysis using cluster analysis of ITS-PCR/*Taq*I products revealed the diversity of the sourdough microbiota, identifying nineteen distinct LAB genotypes. The genotypic diversity of microbial strains appears to be related to the age of the sourdoughs and the influence of the microbiota in the production environment. The highest number of distinct genotypes was observed in rye sourdough samples collected during summer and autumn, likely due to temperature fluctuations in the industrial production rooms. Compared to wheat sourdough, the LAB in rye sourdough were found to be more stable, suggesting that rye flour contributes to microbial stability. Three *L. plantarum* genotypes were consistently found in rye sourdough throughout a year of monitoring, while five genotypes were identified across two seasons. Additionally, LAB with the same genotype were detected in both sourdoughs, indicating microbial transfer between the starters.

This work highlighted that the composition and stability of LAB in two industrial starters can remain consistent over long-term back-slopping and that the industrial environment plays a significant role in the transfer of microbes between sourdough starters.

Understanding the factors that influence sourdough microbiomes deepens our knowledge of microbial ecology, enabling more effective selection of starter cultures and optimization of fermentation conditions. Further research is needed to assess both the technological and functional properties of the isolated strains for their potential use as starter cultures for rye sourdough bread making.

## Figures and Tables

**Table 1:** LAB isolates (n = 66) from rye (RS) and wheat (WS) flour sourdoughs, identified by MALDI-TOF and API^®^ 50 CH kit analysis.

Lactic Acid Bacteria	MALDI-TOF		API Kit	
	WS	RS	WS	RS
*Lactiplantibacillus plantarum*	9	24	9	24
*Lentilactobacillus parabuchneri*	6	2		
*Lacticaseibacillus paracasei*	5	2	5	1
*Levilactobacillus brevis*			2	2
*Limosilactobacillus fermentum*	1	4		5
*Lentilactobacillus buchneri*			4	2
*Lactococcus lactis* ssp. *lactis*			4	1
*Companilactobacillus paralimentarius*	2	1		
*Companilactobacillus mindensis*	3			
*Pediococcus damnosus*			3	
*Leuconostoc mesenteroides* ssp. *cremoris*				1
*Latilactobacillus curvatus*			1	
*Schleiferilactobacillus harbinensis*		1		1
*Weissella cibaria*		1		
*Lactobacillus* sp.	2	1		
*Pediococcus* sp.	1			
Unidentified		1	1	
Total	29	37	29	37

**Table 2:** Genotypic characterization of LAB isolates (n = 61) from rye bread sourdoughs based on ITS-PCR and ITS-PCR/*Taq*I fragment sizes.

Genotype		No. of Isolates	Source of Isolates *	LAB Species	ITS-PCR Sizes (bp)	*Taq*I-Digested ITS-PCR Sizes (bp)
A	A1	4	III RS, IV RS, IV RS, IV RS	*L. plantarum*	530, 440, 280	320, 220, 90
	A2	3	III RS, III RS, IV WS		470, 400, 270	290, 210
	A3	2	IV WS, IV RS		460, 390, 260	400, 340, 240
	A4	3	IV WS, IV RS, IV RS		800, 500, 410, 280	310, 210, 130, 90
	A5	2	I RS, III RS		520, 300	300, 220, 130, 100
	A6	6	I WS, I RS, I RS, III RS, IV RS, IV RS		490, 260	220, 130, 100
	A7	4	I WS, I WS, I RS, I RS		700, 420, 240	700, 160, 100, 80
	A8	2	IV WS, IV RS		530, 280	190, 120, 100
	A9	3	I RS, II RS, III RS		510, 300	400, 200, 180, 120
	A10	1	IV WS		480,250	400, 230
	A11	2	II WS, IV RS		520, 480, 270	230, 120, 100
	A12	1	IV RS		530, 440, 260	300, 200, 170
B	B1	1	II RS	*L. parabuchneri*	670, 530, 420, 260	630, 430, 280, 230, 130, 100
	B2	2	III WS, III WS		570, 480, 280	230, 110, 90
	B3	2	II WS, II WS		720, 440, 280, 240	430, 250, 220
	B4	1	II RS		790, 550, 430, 340, 210, 130	310, 240, 200
	B5	2	IV WS, IV WS		520, 420, 300	280, 220, 180
C	C1	2	II WS, III WS	*L. paracasei*	490, 370, 300, 210	300, 200, 170, 110
	C2	5	III WS, III WS, III WS, III RS, IV RS		500, 250	390, 270, 240, 150
D	D1	1	I RS	*L. fermentum*	490, 400, 300, 270	380, 290, 220, 180, 100, 90
	D2	1	III RS		490, 310	310, 240, 200, 90
	D3	1	II WS		490, 270	340, 220, 130
	D4	1	III RS		510, 440, 370, 280	410, 190, 110, 80
	D5	1	II RS		850, 520, 470, 300	320, 220, 130, 100
E	E1	2	I WS, I WS	*C. mindensis*	420, 210	260, 170
	E2	1	I WS		480, 450, 410, 310, 260	480, 310
F	F1	1	II WS	*C. paralimentarius*	550, 490, 410, 300	380, 280, 200, 180, 110
	F2	2	III WS, III RS		480, 270	330, 220, 200
G		1	III RS	*S. harbinensis*	550, 510, 470, 400, 330, 270	350, 300, 270, 240
H		1	I RS	*W. cibaria*	420, 330, 260	400, 300, 230, 200, 160, 110

* WS—wheat sourdough; RS—rye sourdough; I to IV—sampling periods (I—winter; II—spring; III—summer; IV—autumn).

## Data Availability

The original contributions presented in the study are included in the article, further inquiries can be directed to the corresponding author.
